# Mental health patterns and associated social determinants among university and college students in sub-Saharan Africa during the COVID-19 pandemic era: a scoping review

**DOI:** 10.3389/fpubh.2026.1800724

**Published:** 2026-04-22

**Authors:** Memory Muturiki, Nontsikelelo O. Mapukata, Lawrence Chauke, Sara Jewett

**Affiliations:** 1 Faculty of Health Sciences, University of the Witwatersrand, Johannesburg, South Africa; 2 Division of Public Health Medicine, School of Public Health, University of Cape Town, Cape Town, South Africa; 3 Faculty of Health Sciences, School of Clinical Medicine, University of the Witwatersrand, Johannesburg, South Africa; 4 Health & Society Division, Faculty of Health Sciences, School of Public Health, University of the Witwatersrand, Johannesburg, South Africa

**Keywords:** COVID-19, social determinants of health, social determinants of mental health, student mental health, sub-Saharan Africa, university mental health services

## Abstract

**Background:**

Evidence on student mental health in sub-Saharan Africa (SSA) remains limited, particularly studies examining how mental health patterns intersect with social determinants within higher education institutions (HEIs). This scoping review identifies gaps in the literature documenting student mental health and associated social determinants during the COVID-19 period and highlights priorities for future research in SSA.

**Methods:**

Eight databases (MEDLINE, PsycInfo, Open Access Journals, CINAHL, Google Scholar, Cochrane Library, ProQuest, Scopus) and grey literature were searched for English-language studies from 2020 to 2023. Sixty-seven studies from 214 full-text articles screened met the inclusion criteria.

**Results:**

The included studies varied widely in their examination of student mental health and its links to social determinants of health (SDOH). Mental health was most frequently assessed in terms of depression, anxiety, suicidality, substance use disorders, and psychological distress. Mood disorders were the most commonly reported outcomes. Few studies explored help-seeking behavior. Reported social determinants aligned with structural factors (socioeconomic and political contexts, cultural norms, gender disparities) and intermediary factors such as academic stress, service access, and behavioral patterns including substance use, physical activity, sleep, and diet.

**Conclusion:**

Although many studies addressed social determinants of student mental health in SSA, none provided comparable, multi-country data across HEIs. Most research focused on undergraduate particularly medical students, with limited attention to postgraduate populations. Future work should prioritize multi-country comparative studies and context-specific approaches that strengthen help-seeking and support for at-risk students across diverse SSA settings.

## Introduction

Student mental health refers to the psychological well-being of students, which includes their emotional, cognitive, and social functioning. The World Health Organisation (WHO) describes mental health as “a state of mental well-being that enables people to cope with the stresses of life, realize their abilities, learn well and work well and contribute to their community” (1). In the academic context student mental health is the state of mental wellbeing that enables students to cope with the stresses of life and focus on their academic activities (2). The objective of this scoping review was to explore and map the available literature on mental health patterns and associated social determinants among university students in sub-Saharan Africa (SSA) higher education institutions (HEIs) during the COVID-19 pandemic.

In a study by Ansari and Stock (3), the authors noted that academic stress increased when the demands of academic work exceed students’ coping capacities, leading to physical and emotional strain. Entering university for the first time is a significant transition often associated with high levels of stress as students have to navigate a new social environment. Feelings of isolation and identity uncertainty are more pronounced in students who leave home for the first time (4). Many students in SSA have financial pressures, including tuition costs, living expenses, and the need to balance studies with part-time work (4). These social determinants significantly impact their mental health and overall academic success.

In South Africa, several studies, including those by Bantjes et al. (5) and Makhubela (6), reported on the rise in suicide attempts and complete suicides among students. A Belgium university study has also highlighted a strong association between mental health challenges and academic failure, an important driver of suicidality (7). Depression and anxiety are among the most prevalent mental health issues affecting university students, as reported by Bantjes et al. (8) in South Africa and Shitandi et al. (9) in Nigeria, amongst others. Student mental health is increasingly recognised as a public health concern, because suicide remains one of the leading causes of death among youth aged 15–29 (10).

In South Africa, the university experience is further compounded by structural and historical inequalities that present additional challenges for many students (8). The students considered as more vulnerable tend to come from under-resourced backgrounds, low-resourced schools, low-income households, or rural areas and informal settlements (11). Adjustment difficulties expose vulnerable students to chronic stress, increasing mental health risks and academic problems such as burnout, and diminished performance (12). Similar patterns have been reported among students at resource-constrained universities in low-income countries, including those in SSA (13, 14).

The SSA context is critical because of the complex interplay among structural and intermediary social determinants that influence student mental health in the region. Structural determinants such as widespread economic hardship, under-resourced education systems, and political instability shape HEI students’ access to opportunities and resources creating challenges that place them at greater risk than those in better-resourced regions. Many students in SSA enter university from under-resourced schooling systems and low-income households, placing them at a disadvantage in academic preparation and financial security (8, 11, 13, 15, 16). Inequities in their pre-university education, intersect with intermediary determinants, including living conditions, psychosocial stressors, and access to mental health services. Chronic stress from economic hardship, overcrowded residences, and food insecurity (17) exacerbated by recurrent university strikes or political unrest (18) has an impact on mental wellbeing. Compounding these are cultural stigma, limited mental health literacy, and inadequate institutional support systems, which restrict help-seeking and delay early intervention (19). As such, in the SSA, student mental health must be understood through social determinants lens that reflect the broader conditions affecting university students.

Given the above, systematic reviews have documented a global increase in mental health disorders among university students for longer than a decade (20). However, less is still understood about the specific influences of social determinants on student mental health and the reasons why certain students are more vulnerable to developing mental disorders. According to the WHO (21), social determinants of health (SDOH) encompass the conditions in which individuals are born, live, study, and carry out daily activities. These determinants include behavioral factors such as cigarette smoking, alcohol consumption, and substance use which are linked to poor mental health outcomes in students (22, 23). This scoping review is part of a doctoral degree using a conceptual framework adapted from the WHO’s Conceptual Framework for the Social Determinants of Health (24) to describe social determinants of students’ mental health in the South African context. Student mental health difficulties in the SSA region are often linked to intersecting SDOH such as first-generation status, financial challenges, food insecurity, academic pressure, and disadvantaged backgrounds (7, 8, 11, 25–28).

The COVID-19 pandemic, which began in 2020, further exacerbated mental health challenges among university students globally including those in sub-Saharan African countries. International studies have consistently reported significant increases in psychological distress during the pandemic, with heightened levels of anxiety, depression, and stress among students (29–32). The WHO estimated the prevalence of mental illness in young adults is 20–25% (33), however COVID-19 pushed this figure close to 30% (10). The COVID-19 pandemic brought student mental health challenges to surface, largely due to the disruptions caused by lockdown measures (34). The rapid migration to online learning, platforms, campus closures, and social distancing measures drastically changed students’ academic and social environments (35–37). Universities have been advised by several authors in SSA to respond to the impact of COVID-19 by incorporating targeted support strategies for vulnerable student populations (25, 38, 39). In particular, the association of social determinants with mental health among students requires careful attention in the design of institutional responses (26). A rapid review of student mental health services in SSA found that the pandemic did not create new challenges but intensified existing problems rooted in economic hardship, under-resourced institutions, and limited mental health services (40). Similar challenges have been described in studies by several authors based in SSA region (41–43). Given the documented impact of the COVID-19 pandemic on mental health services within public health systems (34) and the corresponding rise in psychological distress among university students (35) research is needed to further elucidate the impact of social determinants of health on the mental health patterns among university students in SSA.

Most research on student mental health comes from the Global North, leaving limited understanding of how it is conceptualised within the SSA, especially regarding relevant social determinants. The SSA region presents a unique set of complex socio-political and economic challenges that have implications for student mental health (13). Several authors (17, 25, 38, 39) have pointed out that there is a critical need for context-specific research and targeted policy initiatives to strengthen mental health services that reflect the social, cultural, and institutional dynamics of African HEIs. Considering the current gaps in the literature, this scoping review aimed to address the following research question: What are the existing knowledge gaps in how student mental health is framed, and which social determinants have been explored among students in HEIs in SSA during the COVID-19 era?

## Methods

This scoping review was conducted and reported in accordance with the methodological framework outlined in the Preferred Reporting Items for Systematic Reviews and Meta-Analyses extension for Scoping Reviews (PRISMA-ScR) guidelines, as proposed by Peters et al. (44). We focused on student mental health during the COVID-19 era, with particular attention to the associated social determinants. We utilised a systematic approach to identify, analyse, and map patterns in student mental health and related social determinants, while also highlighting gaps in the literature from geographic, methodological, and population-level gaps. This scoping review includes studies from all types of HEIs in which students reported experiencing mental health challenges, those with diagnoses of mental disorders, accessing mental health or student support services, or encountering difficulties related to student wellbeing and coping with life stresses. Eligible settings encompassed, but were not limited to, public, private, and non-governmental universities and colleges across SSA. The only African countries that are not in the sub-Saharan region are Algeria, Egypt, Libya, Morocco, and Tunisia. Studies from these countries were excluded.

### Inclusion and exclusion criteria

We used the participant, concept, context (PCC) framework for eligibility criteria as recommended by Peters et al. (44), Table 1.

**Table 1:** Participant, concept, context (PCC) framework for eligibility criteria.

Inclusion	Exclusion
Participants	
Participants were students enrolled in HEIs (post-school universities and colleges)	Studies not focused on students in HEIs or not about students
	Studies on students and non-students but which did not disaggregate data for students
Concept	
Studies which examined both student mental health and the association with social determinants	Studies that focused exclusively on either mental health or SDoH without examining their interrelation
Empirical studies quantitative, qualitative, or mixed methods	Secondary literature (e.g., narrative, scoping, or systematic reviews)
Peer-reviewed literature, and grey literature (only postgraduate dissertations)	Study protocols, preprints, commentaries, and opinion pieces
Context	
HEIs (public, private, and non-governmental) universities and colleges across sub-Saharan Africa	School-level studies
	Studies published outside SSA
Studies published in English during the study period (2020–2023)	
	Multi-country studies on SSA countries and countries outside the geographic region which did not disaggregate for countries in SSA
	Studies published in languages other than English

Participants were students enrolled in HEIs, defined as post-school universities and colleges. The concept focused on student mental health and its association with SDOH. The context was sub-Saharan African HEIs during the COVID-19 pandemic period. Inclusion was limited to empirical quantitative, qualitative, or mixed methods studies published in English between 2020 and 2023, corresponding to the COVID-19 outbreak years. In addition to peer-reviewed literature, relevant grey literature such as postgraduate dissertations were also considered where they provided insights aligned with the review objective and research question. Eligible studies were conducted within SSA and examined both student mental health and the association with social determinants.

We excluded studies conducted outside SSA, those not focused on students in HEIs, and documents published before 2020 or in languages other than English. Primary school-level studies, as well as documents that focused exclusively on either mental health or SDOH without examining their interrelation, were also excluded. In addition, secondary literature (e.g., narrative, scoping, or systematic reviews), study protocols, preprints, commentaries, and opinion pieces were not considered. Documents were excluded if they were published outside the study period, were not focused on the specified geographic region, or did not pertain to students. Articles published within the study period but based on data collected before the onset of the COVID-19 pandemic were excluded, as their findings did not align with the study’s research question.

### Search strategy

Our search strategy aimed to identify both published and unpublished relevant studies, conducted in consultation with a research librarian. We initially started by conducting a limited search of MEDLINE (PubMed) and PsycInfo to identify availability of relevant documents. Appendix A (Supplementary File) illustrates the index terms used in the initial search strategy.

We then used the index terms to develop a full search strategy, Appendix B (Supplementary File). This was adapted for each database in line with Wits-JBI evidence synthesis method (44). The reference list of all included and excluded studies were then screened for additional studies. The databases searched were MEDLINE (PubMed), PsycInfo, EMBASE, Open Access Journals, CINAHL (EBSCOhost), and ProQuest, Google Scholar, the Cochrane Library (Ovid), and Grey Literature (postgraduate dissertations).

### Study/source of evidence selection

**Figure 1:**
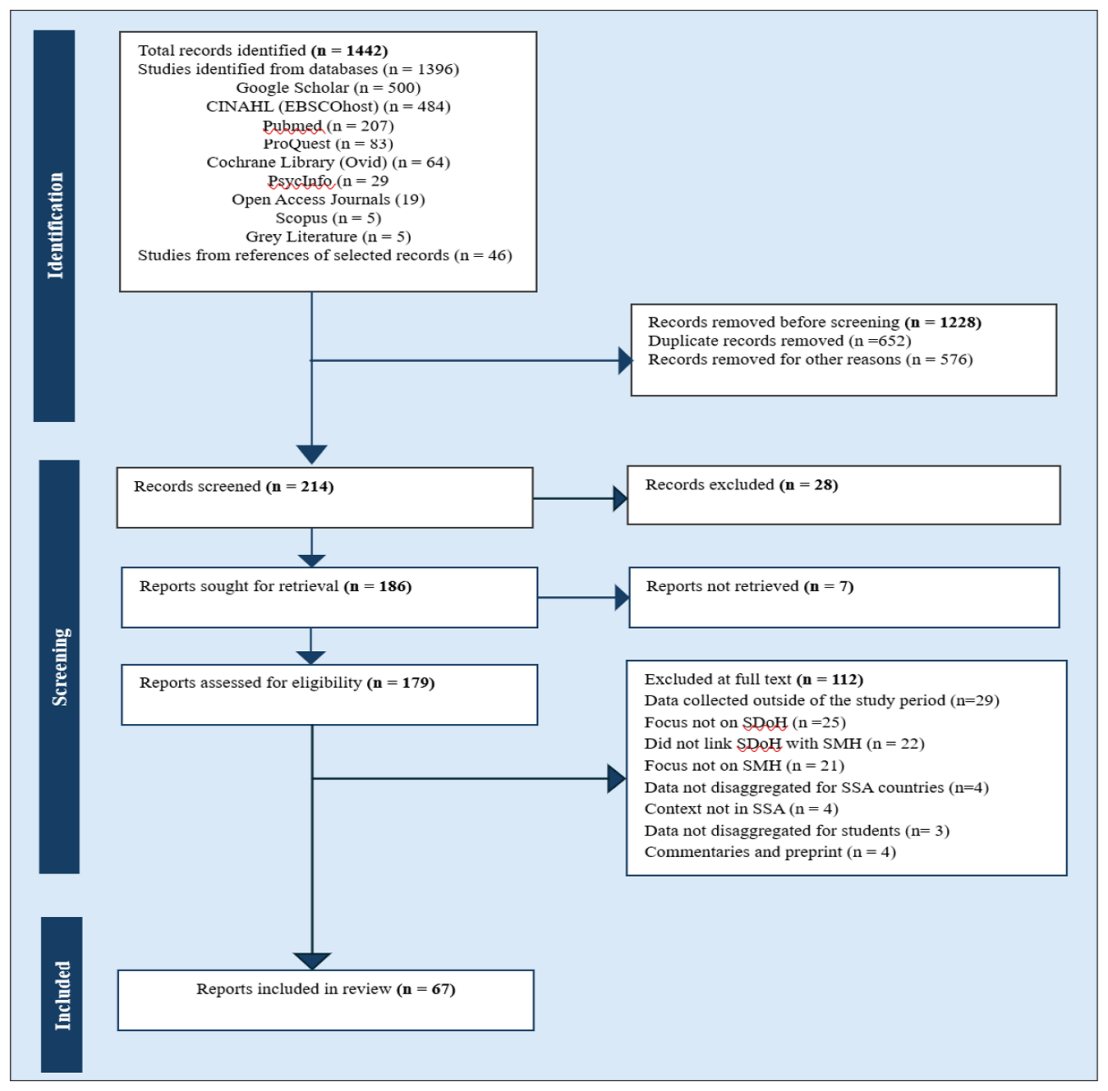
PRISMA flow chart of the study selection process adapted from Page et al. (45).

Following the search, all identified citations were collated and uploaded into EndNote 21 (Clarivate Analytics, PA, USA) and duplicates were removed. An initial pilot screening was conducted, during which the titles and abstracts of all identified documents were independently reviewed using the predefined inclusion criteria. Documents that met these criteria were retrieved in full and imported into NVivo 15 for data management and analysis. The full-text articles were subsequently evaluated for eligibility. Reasons for exclusion at the full-text review stage were documented and are reported in the results (Figure 1). The overall search and study selection process is comprehensively reported and presented below using the PRISMA-ScR flow diagram (45). The reference lists of the selected studies, as well as those that were not selected, were further explored to identify additional studies meeting the eligibility criteria, an additional twenty-four articles were identified through this process.

### Screening and data extraction

A data extraction tool was developed by the reviewers and used for data collection, Appendix C. Specific details about the participants, concept, context, study methods, main data on student mental health and the associated SDOH as well as gaps in reporting were extracted in line with the research question. There were no additional modifications made to the data extraction form. Two reviewers (MM & NM) independently screened and reviewed records. They compared their decisions and resolved any discrepancies through discussion. A third reviewer (SJ) was consulted when needed to reach consensus. No formal inter-reviewer agreement statistic was calculated. MM further searched the included and excluded articles’ reference lists and extracted 24 more articles and the second and third reviewers (NM and SJ) confirmed accuracy of the data extraction. We extracted the following data from the included documents: (i) study and sample characteristics [authors, year of publication, country of study, population targeted (HEIs students), sample demographics, purpose of study, study methods, sample size], (ii) student mental health association with SDOH, (iii) key findings and gaps in reporting.

### Data analysis and presentation

Using the data abstraction tool data were extracted and results synthesized and reported in accordance with PRISMA-ScR (46). Figure 1 below is the PRISMA flowchart summarising the study selection process. After screening full-text articles we used NVivo-15 to code and generate themes.

## Results

As summarised in Figure 1, a total of 67 documents addressing student mental health and its associated social determinants in sub-Saharan Africa (SSA), with data collected and published during the COVID-19 pandemic period (2020–2023). Among these, five were postgraduate dissertations representing grey literature, while the remaining studies were drawn from peer-reviewed journal articles. There were no special cases included.

### Study details

**Figure 2:**
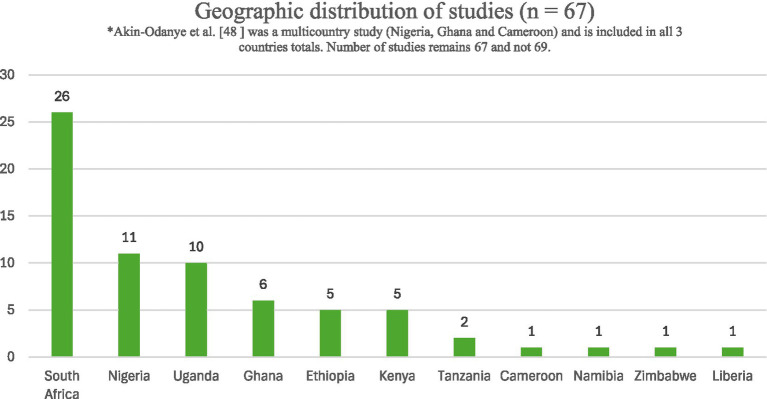
Geographic distribution of the studies which met the inclusion criteria.

Geographically (Figure 2), the studies were conducted in 11 countries with the majority (70.2%, *n* = 47) of these studies being from only three countries in SSA (South Africa, Nigeria and Uganda). More than half of studies (*n* = 48) reported on the relationship between gender and student mental health while another 31 studies were dedicated to medical undergraduate cohorts. Only two studies were multi-country studies with other countries outside SSA (30, 47) and one was a multi-country study which collected data from the US and three countries in SSA, these being Cameroon, Ghana and Nigeria (48).

**Figure 3:**
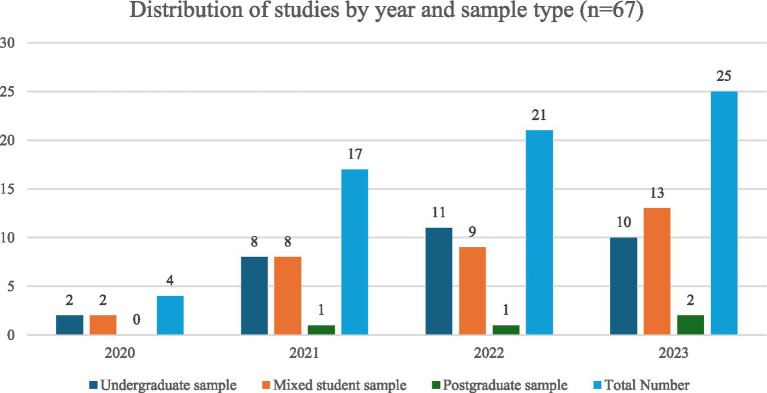
Distribution of included studies by year of publication and student sample type.

Figure 3 below illustrates studies that were published on student mental health and the associated SDOH over the four-year study period. Most of the included studies (76.1%, *n* = 51) were cross sectional surveys. Eleven studies (16.4%) used qualitative methods, only three studies incorporated a mixed-methods approach, while one study by Eseadi (49) used a randomised controlled trial design, and one was a secondary data analysis (50).

### Populations targeted

The selected empirical studies recruited just over 89,000 students with varying sample sizes ranging from 9 to 28,516. One study combined students and non-students (51) and another HEIs and high school learners (52); both were included because the results for HEIs students in these studies were disaggregated. Almost a third of the studies examined medical and health students (26.9%). In terms of the study program most studies focused on undergraduate students (49.3%) or included both undergraduate and postgraduate students (43.1%). Only 4 (6%) (54, 67, 127, 128) studies published on only postgraduate students.

### Social determinants of health

According to the WHO (2), social determinants of mental health are a subset of SDOH, specifically focusing on factors that affect mental health and psychological wellbeing. The social determinants of student mental health reported in the included studies, aligned with the WHO Conceptual framework are listed in Table 2. When individual studies addressed multiple social determinants, they were classified into more than one category. Therefore, the total counts and frequencies for these determinants exceed the number of studies included.

**Table 2:** Structural and intermediary determinants of student mental health examined.

Social determinants of health		Description	Number of studies (*n*)
Structural	Gender	Socially constructed roles and inequalities	30
	Socioeconomic and political context	Macroeconomic, cultural and policy environment	13
	Societal norms and cultural beliefs	Societal norms, cultural beliefs, spiritual, religious, stigma and beliefs around mental health	11
Intermediary	Psychosocial factors	Academic stress, year of study, degree program, other student-related challenges	49
	Social and family relationships	Family support, social cohesion, peer networks	17
	Material circumstances	Financial resources and living conditions	18
	Access to mental health services	Availability, affordability, acceptability of mental health care	16
	Behavioral factors	Coping behaviors, help-seeking patterns, substance use	20

### Documented student mental health patterns

Across studies, the most frequently reported student mental health disorders were depression and anxiety-related conditions. Table 3 illustrates the identified student mental health patterns across SSA countries.

**Table 3:** Student mental health patterns reported with social determinants across the study period (2020–2023).

Mental disorders examined		2020	2021	2022	2023	Total	Countries and number of studies (*n*)
Suicidality (*n* = 12)	Suicide	0	1	1	0	2	Uganda (2)
	Suicide attempt	0	1	1	1	3	Uganda (1), Nigeria (2)
	Suicide ideation	0	1	1	5	7	South Africa (3), Nigeria (3), Uganda (1)
Mood disorders (*n* = 40)	Anxiety related disorders (GAD, PTSD, panic disorder, SAD)	1	7	3	8	18	Nigeria (2), South Africa (7), Uganda (1), Ethiopia (3), Ghana (2), Kenya (1), Tanzania (1), Namibia (1)
	Depression	4	10	3	4	21	South Africa (6), Nigeria (4), Uganda (2), Ethiopia (3), Ghana (1), Liberia (1), Kenya (2), Tanzania (1), Namibia (1)
	Bipolar mood spectrum	0	0	0	1	1	South Africa (1)
Substance use disorders (*n* = 9)	Alcohol use disorder	0	2	1	2	5	South Africa (1), Nigeria (2), Kenya (2)
	Substance use disorder	0	2	1	1	4	South Africa (1), Nigeria (1), Kenya (2)
General mental health (*n* = 39)	Psychological distress	0	2	4	4	10	Cameroon (1), Ghana (2), Nigeria (5), Uganda (1), South Africa (1)
	General psychiatric disorders	1	0	2	0	3	Nigeria (2), Kenya (1)
	General mental health status	0	4	9	13	26	South Africa (16), Tanzania (1), Ethiopia (1), Uganda (4), Nigeria (1), Ghana (1), Kenya (1), Zimbabwe (1)

Mood disorders were described in 40 studies, with major depressive episode (MDE)/depression featuring prominently (*n* = 21), and one study reporting on the bipolar mood spectrum. Anxiety-related disorders were identified in 18 studies, including generalised anxiety disorder (GAD), social anxiety disorder, post-traumatic stress disorder (PTSD), and panic disorder. General mental health concerns (category, reflecting broad psychological difficulties without specific diagnoses) were reported in 39 studies, including psychological distress (*n* = 10), general psychiatric disorders (*n* = 3) and general mental health status (*n* = 26). Suicidality was addressed in 12 studies, with suicide ideation (*n* = 7), suicide attempts or self-harm (*n* = 3) and completed suicide (*n* = 2) being reported. Substance use disorders were less commonly examined, with only 10 studies focusing on drug use disorder (*n* = 4) (25, 43, 53, 54) and alcohol use disorder (*n* = 6) (25, 43, 53–56) however, as many as nine other studies (51, 57–64) mentioned alcohol use in relation to demographics, lifestyle and coping mechanisms of the participants who presented with mental symptoms. These findings highlight the predominance of depression and anxiety among students, alongside general psychological distress and concerning levels of suicidality.

### Themes

#### Structural determinants of student mental health

This theme encompassed broader societal factors that influence the unequal distribution of power, income, and resources, which in turn affect mental health outcomes among students (24), Table 4.

**Table 4:** Distribution of studies by WHO SDOH framework.

SD of student mental health		Studies	Total number of studies (*n*)
Structural	Gender	Biological and socially constructed roles and inequalities, gender-based disparities in mental health prevalence help-seeking behavior, and exposure to risk factors (4–6, 9, 25, 39, 42, 43, 47, 58, 61, 62, 66, 68, 72, 74, 75, 78, 80, 83, 84, 93–101)	30
	Socioeconomic and political context	Macroeconomic, cultural and spiritual beliefs, and policy environment, focusing on macro-level conditions such as national policies, social inequalities, and institutional responses that impact student wellbeing (17, 19, 25, 30, 43, 48, 54, 58, 71, 98, 102–104)	13
	Societal norms and cultural beliefs	Societal norms, cultural beliefs, indigenous spiritual practices, religious, stigma, and beliefs around mental health (19, 51, 60, 79, 94, 105–109)	11
Intermediary	Psychosocial factors	Academic stress, year of study, degree program, other student-related challenges, general psychological stress, sleeping patterns, university institutional culture (4, 9, 14, 16, 39, 47, 48, 50–55, 57–61, 63, 64, 66, 68, 71, 75, 76, 80, 83, 91, 93, 95, 96, 101–103, 106, 109–122)	49
	Social and family relationships	Family support, social support, peer networks (4, 30, 50, 51, 54, 57, 74, 95, 100, 107, 108, 111, 114, 120, 122–124)	17
	Material circumstances	Financial resources, food insecurity and living conditions, difficult early life experiences, family environment, peer relationships, social networks, food security, availability of housing, access to education (30, 37, 50, 54, 57, 59, 71, 74, 76, 98, 100, 102, 104, 107, 109, 111, 116, 123)	18
	Access to mental health services	Availability, affordability, acceptability of mental health care, help-seeking patterns, campus mental health services (19, 37, 51, 53, 67, 95, 99, 103, 105–108, 112, 115, 125, 126)	16
	Behavioral factors	Coping behaviors, substance use, smoking cigarettes, use of recreational drugs such as cannabis and drinking alcohol (25, 43, 50, 51, 53–60, 62, 66, 74, 84, 91, 107, 109, 110)	20

Gender was the most frequently reported structural determinant, identified in 30 studies. These studies explored gender-based disparities in mental health prevalence, help-seeking behavior, and exposure to risk factors. Socioeconomic and political context was examined in 13 studies, focusing on macro-level conditions such as national policies, social inequalities, and institutional responses that impact student wellbeing. Societal norms and cultural beliefs were reported in 11 studies, reporting on how traditional values, stigma, and beliefs around mental health and gender roles influence both the perception and uptake of mental health services.

### Intermediary determinants of student mental health

This theme covered the immediate and proximal conditions in which students live, study, and interact factors that directly influence mental health outcomes (2). The theme appeared many times across the included studies as intermediary determinants of student mental health, Table 4. Social support was reported in 17 studies, emphasizing the protective role of peer relationships, family networks, and institutional support systems in buffering psychological distress and promoting resilience. Financial challenges experienced by students emerged as a significant stressor described in 19 studies, with common concerns including tuition fee burdens, lack of study funding, and cost of living. Material circumstances, including housing conditions, food insecurity, and access to learning resources, were also addressed in 18 studies. Student-related psychosocial factors, such as academic stress, study year, and programme type, appeared in 49 instances across the studies. Behavioral factors were examined in 20 studies, including substance use, sleep patterns, physical activity, and coping mechanisms. These behaviors were often shaped by underlying structural stressors and had significant impact on students’ mental wellbeing. The mental health impact of the COVID-19 pandemic was a described in 25 studies, reflecting its significant contribution to student psychological distress across the region. The most described issues included anxiety, isolation, academic disruptions, online and remote learning difficulties, and reduced access to in-person mental health services. Spiritual wellbeing was explored in 4 studies, where faith-based coping, religious identity, and indigenous spiritual practices were found to influence and help-seeking attitudes.

### Digital and social media influences

Though not explicitly named in the WHO’s SDOH framework, two studies examined the relationship between social media use and student mental health. Both highlighted the dual impact of social media providing peer connection and access to support, while also contributing to anxiety, low self-esteem, and digital fatigue. The findings point to the need for balanced digital engagement and the promotion of digital wellbeing as part of broader mental health strategies.

### Access and utilization of mental health services among students

One fifth of the studies (*n* = 16) addressed the domain of access to and utilization of mental health services among university and college students. This thematic area is included in Table 4. First, knowledge and perceptions of mental health were examined in 4 studies, highlighting varying levels of awareness, stigma, and attitudes that influence service uptake. Second, help-seeking behavior was the focus of 8 studies, with findings pointing to a range of individual, cultural, and structural factors that facilitate or hinder students’ utilisation of mental health services. Third, digital and online mental health interventions were examined in 9 studies, reflecting growing interest in the role of technology such as mobile applications and online counselling platforms in improving access, especially during periods of remote learning. Fourth, traditional therapeutic interventions, including in-person counselling services, were covered in 3 studies, with emphasis on availability, student satisfaction, and barriers such as limited capacity. The gaps identified in this study (see Table 5).

**Table 5:** Summary of gaps in reporting identified.

Gaps identified in describing student mental health and the associated social determinants
General terms on framing student mental health
Over-reliance on general terms, “Psychological Distress, Mental Distress” was used to in many studies describe mental health symptoms Few studies used structured clinical assessments and limited use of standardised diagnostic tools Most studies relied on screening tools (PHQ-9, GAD-7), with limited validation in African contexts
Cultural conceptualisations of mental health
Few studies examined how student mental health is culturally understood or expressed within diverse sub-Saharan African HEI contexts
Gaps in explored social determinants
Glaring gap was historic context and its impact in educational systems across the region Early life adversities were mentioned in some studies but rarely examined as mediators or moderators of mental health Peer networks were mentioned with little data on the protective or risk-enhancing roles of peer relationships Few studies explored the mechanisms of how behaviors such as alcohol use, smoking, or cannabis use are linked to mental health among students Social media exposure is an emerging determinant of distress or resilience, yet few studies explored it None of the studies explored climate change and mental health
Service access and health system gaps
Limited data on actual help-seeking practices and preferences among students Structural mental health system challenges, including provider shortages were noted in few studies and lack comparability across SSA
Geographic
Data was only from 11 of 46 countries in SSA Over representation of a few countries (South Africa, Nigeria and Uganda), with under representation other countries in SSA
Methodological
Limited longitudinal or comparative designs Most studies (87%) are cross-sectional Very few studies involved qualitative methods to capture lived experiences or preferences of students

## Discussion

This scoping review maps how student mental health is framed in the literature and which social determinants have been examined among students in SSA higher-education setting during the COVID-19 and identifies the social determinants under study. It shows consistently high rates of depression, anxiety and psychological distress, often examined in isolation among students (14, 25, 42). Although several social determinants were addressed in the studies, such as academic stress, gender, socioeconomic challenges, and limited access to mental health services, most authors explored them in isolation rather than through an integrated lens. Moreover, significant knowledge gaps persist regarding the mental health experiences of postgraduate students and students from underrepresented regions in SSA.

Our findings identified several recurrent mental health patterns among university and college students across SSA during the COVID-19 era (Table 3). Depression and anxiety emerged as the most frequently reported disorders, commonly assessed using validated screening tools such as the PHQ-9 and GAD-7. These findings align with prior systematic reviews that have documented rising rates of mental disorders in higher-education institutions over the past decade, even before the pandemic (20, 65).

The conceptualisation of student mental health in SSA varies across studies. Several authors (42, 49, 58, 66–68) grouped a spectrum of mental well-being symptoms under broader categories such as psychological distress or emotional well-being. These reflected subclinical or undiagnosed symptoms that impair daily functioning and academic performance. Bantjes et al. (25) similarly emphasise that these patterns underscore the need for early detection and support systems within HEIs.

Emerging reports of suicidality, including suicidal ideation and suicide attempts, highlight serious mental-health risks exacerbated by the pandemic and its socio-economic effects (38, 39, 48). Other commonly reported challenges such as stress, and academic-related difficulties suggest that academic pressure remains a major source of psychological distress for students. Despite these recurring themes, few studies explored help-seeking behaviors, revealing a substantial gap in understanding how students access and utilise mental-health services, their preferences, and the role of informal support systems. Several authors (8, 12, 15–17, 38, 39, 129) agree that these patterns collectively indicate an urgent need to scale up contextually relevant, accessible, and evidence-based interventions tailored to SSA student populations.

Country-specific findings in SSA further illustrate this trend. In Ethiopia, Aylie et al. (41) reported a 21.3% prevalence of depression during the early pandemic phase slightly higher than the 15% prevalence observed in South Africa by Bantjes et al. (25). In Nigeria, students reported increased emotional distress and academic disruption, compounded by limited access to online learning and mental-health support (42). In Kenya, Mutiso et al. (43) highlighted how pre-existing mental-health and socio-economic vulnerabilities were intensified by pandemic-related uncertainty and restricted access to counselling services.

In this study, we described structural determinants of student mental health described that aligned with the WHO Framework including governance, public policy, cultural norms, and socioeconomic factors such as income inequality and educational access (21). As reported by Ataguba et al. (69), students are a microcosm of the community and as such, student mental health has been reported by several authors to be influenced by structural forces, including social networks, higher education policies, and funding systems (4, 25, 70).

In South Africa, initiatives like government funded student bursaries aim to reduce inequities by supporting vulnerable students from disadvantaged backgrounds (32). However, this review found that while financial challenges were frequently noted as social determinants, few studies critically examined the role of national and institutional policies in shaping student wellbeing. The plight of vulnerable students is more pronounced as their families, are mostly affected by the current economy and food insecurity (26, 71), making it difficult to adequately support their children’s studies. In South Africa, there is evidence of the historic apartheid system responsible for the ongoing inequalities (72) but the impact of colonial history was not reported in any of the other studies despite colonial rule that prevailed in the African continent.

Most studies (Table 4) reported on increased mental disorders in female participants, supporting what is already known in the literature (42, 66, 73); however, none of the studies examined the role of gender and its influence on education outcomes. While several studies reported higher prevalence of mental disorders among female participants (42, 66, 74), this aligns with existing literature reports that young women are at higher risk for anxiety, depression, and other common mental disorders. For example, female students in SSA experience academic pressure due to competing household responsibilities, financial dependence, and gendered expectations within their families (4, 9, 73) and for some undergraduate students, Alabi et al. (74) cited unplanned pregnancies as another factor. A study in Kenya by Aloka (4) reported that gender was significantly associated with adjustment challenges among first year students, with female students experiencing greater difficulties compared to their male counterparts. Other studies such as Chigerwe et al. (47) however reported no significant differences in prevalence of mental disorders between males and females. Experiences of gender-based violence (GBV), history of sexual assaults on and off campus, and safety concerns particularly in females were associated with mental disorders described mentioned in several studies across SSA (50, 55, 74–76). COVID-19 pandemic exacerbated the socioeconomic status of many students taking a toll on their mental health (65, 77).

Several authors (64, 78–80) described barriers to student mental health service access, including cultural and belief systems, stigma, societal attitudes towards help-seeking, and a preference for alternative forms of treatment. These findings highlight the complex interplay between structural inequalities, daily stressors, and student-specific challenges that shape student mental health. Aligned with Sustainable Development Goal 3 (SDG 3) on good health and well-being (81), this review supports the global agenda to promote mental health and reduce health disparities by providing evidence on the social determinants affecting mental well-being among students in the SSA region.

Alcohol use emerged as a recurring intermediary determinant of student mental health, with 12 studies specifically examining this issue. Several studies reported strong associations between alcohol or substance use and depressive symptoms, as well as suicidality (51, 55, 56, 64, 82, 83), while two studies reported no significant association between substance use and depression. For instance, Shah et al. (54) found no link between depression and substance use, while Kaggwa et al. (84) reported that none of the students who died by suicide in their study were reported to have had substance dependence. In studies that explored alcohol use, most participants confirmed using alcohol as a form of self-medication to manage emotional distress or to temporarily improve their mood (25, 43, 53, 58). These findings reinforce existing literature suggesting a bidirectional relationship, where alcohol use may both result from and contribute to mental disorders (85–87). Across most studies, alcohol was the most frequently abused substance among students (43, 54, 58, 64, 83).

Among students in HEIs, factors like psychological stress, disrupted sleep patterns, academic pressure, and institutional culture were described by several studies as contributing to mental health outcomes (59, 60, 62). The findings of this study confirm what has been reported by other authors (88, 89) about the historic and structural inequities across SSA that continue to shape the social determinants of student mental health, particularly for low-income families and students of rural origin.

Even though many HEIs in South Africa are reportedly offering mental health services on campus at low or no cost, students frequently face various access barriers (82). A regional systematic review by Komu et al. (90) described widespread poor mental health literacy together with cultural and religious beliefs as contributing to barriers in professional help-seeking, especially in rural areas. Linked to this, the findings of this review revealed a paucity of culturally relevant studies that explore how university students in SSA understand, express, or internalise their mental health. This represents a significant knowledge gap, given that according to Pederson et al., cultural beliefs and norms shape how students in the region perceive mental health, interpret symptoms, and engage in help-seeking behaviors (79). Moreover, the limited focus on help-seeking behaviors in this study highlights the need for contextually appropriate and student-acceptable strategies to improve access and support in HEIs.

Despite limited resources, digital and social media usage are emerging as significant factors in shaping mental health awareness and conversations across SSA. In the region, stressors that students face include challenges in internet connectivity and access to data as part of the larger socioeconomic and political context. Although only two studies (91, 92) addressed this, both indicated the bidirectional relationship of social media usage in students’ mental health. While digital platforms were described as offering options for connection and support, they also contributed to increased anxiety, depression and digital fatigue (91). These findings call for more targeted research and the promotion of digital well-being within student mental health strategies.

### Strengths and limitations

A key strength of this study is its methodological rigor and comprehensive scope achieved by using the JBI guide, which included systematic searches across six major databases as well as an extensive search of the grey literature. Another notable strength lies in the study’s focus on timely research in the COVID-19 era using a social determinants lens within a geographical context where evidence synthesis is much needed to address university student mental health patterns.

This review also has limitations worth mentioning. Firstly, seven documents with relevant titles and abstracts could not be retrieved in full, potentially leading to the exclusion of important data. The inability to access these full texts introduces a risk of publication or selection bias, as the unavailable studies may have differed systematically in their findings or methodological quality. Secondly, the review was restricted to documents published in English. This is a significant limitation given the language diversity of SSA, which includes 21 French-speaking countries and six Lusophone (Portuguese-speaking) countries. As a result, studies published in French or Portuguese, conducted in higher-education institutions within these regions were not captured. This language restriction may have led to the underrepresentation of mental health experience from West, Central and parts of Southern Africa, thereby limiting the geographical, cultural and contextual breadth of the evidence base. Moreover, excluding non-English studies may have contributed to a bias toward countries with stronger anglophone research infrastructures, further skewing the findings toward already well-represented contexts such as South Africa, Nigeria and Uganda. Additionally, limiting the search to English-language publications may have overlooked culturally grounded conceptualisations of mental health that are more likely to be documented in local languages. Together, these limitations point to the need for future reviews that include multilingual search strategies, broader regional representation, and mechanisms to access hard-to-retrieve documents to ensure a more comprehensive understanding of student mental health across the SSA region.

## Conclusion

Given the purpose of this scoping review, the findings reaffirm the growing mental health burden among university students, shaped by both structural and intermediary determinants. In this paper, we demonstrated that student mental health in sub-Saharan Africa is shaped by a complex interplay of structural, cultural, and intermediary social determinants that were exacerbated further during the COVID-19 pandemic. This review underscores the urgent need for coordinated policy action and institutional investment to strengthen campus-based mental health support, guided by equity, contextual relevance, and evidence. Advancing research and innovation particularly through digital and data-driven solutions will be essential to achieving sustainable improvements in student wellbeing across the region. Of significance is that across the 67 included studies, recommendations consistently called for policy-level interventions and institutional commitment to strengthen student mental health systems. Universities and policymakers should prioritise the development of accessible, comprehensive, and context-sensitive mental health services, particularly for students at heightened risk due to social and economic vulnerabilities. Institutional strategies should integrate culturally responsive approaches, equitable academic support, and targeted interventions that reflect the diversity of student populations in the region.

Future research should directly investigate how specific social determinants of health shape student mental health with particular attention to emerging factors such as climate change and the growing influence of artificial intelligence on wellbeing. There is a need for comparative, multi-country studies across higher-education institutions in different SSA to generate regionally comparable data and address persistent gaps in underrepresented settings. Research must also prioritise postgraduate students, whose mental health remains poorly documented, and further examine help-seeking behaviors and barriers to accessing mental health services. Additionally, studies should evaluate the effectiveness, acceptability and scalability of digital mental-health interventions, including AI-enabled tools and mobile applications, to strengthen capacity in resource-constrained environment and expand early identification and support for at-risk students.

## Data Availability

The original contributions presented in the study are included in the article/Supplementary material, further inquiries can be directed to the corresponding author.
