# Supplementary material for: Mental health patterns and associated social determinants among university and college students in sub-Saharan Africa during the COVID-19 pandemic era: a scoping review
Source: Front Public Health. 2026 Apr 22;14:1800724. doi: 10.3389/fpubh.2026.1800724 (PMC13143764; doi:10.3389/fpubh.2026.1800724)
Supplement: Supplementary file 1 [file Table_1.DOCX]

**SUPPLEMENTARY MATERIALS**

**Appendix A: Initial Search Strategy**

| **Concept** | **Context** |
| --- | --- |
| Student mental health  or  Mental health  or  Mental disorders  or  Mental illness  or  Psychiatric disorders  or  Mental wellbeing  or  Student wellness  or  Social determinants  or  Social determinants of health  or  Social determinants of mental health  or  COVID-19 era  or  COVID-19 pandemic  or  COVID-19 outbreak period | University healthcare services  or  University health services  or  College health services  or  University mental health services  or  Campus-based health services  or  Sub-Saharan Africa universities  or  Campus-based clinics  or  College students  or  University students  or  Sub-Saharan Africa Higher Education Institutions  or  Africa |

**Appendix B: Search**

| **Concept** | **Search Keywords** | **Records Retrieved** |
| --- | --- | --- |
| 1.Mental health | “Student mental health” OR “Mental health” OR “Mental disorders” OR “Mental illness” OR “Psychiatric disorders” OR “Mental wellbeing” OR “mental health pattern” OR “Student wellness” OR “wellbeing” OR “wellness” |  |
| 2.Social determinants | “Social determinants” OR “social determinants of health” OR “Social determinants of mental health” |  |
| 3.HEI student | “Universities and colleges” OR “Higher Education Institutions” OR “University healthcare services” OR “College health services” OR “University mental health services” OR “Campus-based health services” OR “Sub-Saharan Africa universities” OR “Campus-based clinics” OR “College students” OR “University students” OR “post school” |  |
| 4.COVID-19 | “COVID-19 era” OR “COVID-19 outbreak period” OR “COVID-19 pandemic” OR “COVID-19” OR “sars-cov-2” OR “pandemic” |  |
| 5. SSA | “SSA” OR “Sub-Saharan African” OR “Africa” |  |
| 6. | 1 and 3 |  |
| 7. | 2 and 3 |  |
| 8. | 1, 3 and 4 |  |

**Appendix C: Data Extraction Tool**

| **Authors** | **Year of Publication** | **Country** | **Population** | **Sample Demographics** | **Purpose of research** | **Methods** | **Mental health association with SDOH** | **Context of study**  **(HEIs)** | **Key Findings** | **Gaps in reporting of project** |
| --- | --- | --- | --- | --- | --- | --- | --- | --- | --- | --- |
|  |  |  |  |  |  |  |  |  |  |  |
|  |  |  |  |  |  |  |  |  |  |  |
|  |  |  |  |  |  |  |  |  |  |  |
|  |  |  |  |  |  |  |  |  |  |  |

**Appendix D: Studies Meeting Criteria**

| 1. Adjepong M, Amoah-Agyei F, Du C, Wang W, Fenton JI, Tucker RM. Limited negative effects of the COVID-19 pandemic on mental health measures of Ghanaian university students. Journal of Affective Disorders Reports. 2022 Jan 1; 7:100306. 2. Agyekum B. Adult student perspectives toward housing during COVID-19. Wellbeing, Space and Society. 2022 Jan 1;3:100086. 3. Akin-Odanye EO, Kaninjing E, Ndip RN, Warren CL, Asuzu CC, Lopez I, Muiruri C, Vilme H. Psychosocial impact of COVID-19 on students at institutions of higher learning. European journal of education studies. 2021;8(6):112. 4. Alabi, A.A., Oladimeji, O.K. and Adeniyi, O.V., 2021. Prevalence and factors associated with suicidal ideation amongst college students in the Nelson Mandela Bay Municipality, South Africa. South African Family Practice, 63(1). 5. Ali SK, Shah J, Du K, Leekha N, Talib Z. Mental health disorders among post graduate residents in Kenya during the COVID-19 pandemic. Plos one. 2022 Apr 4;17(4):e0266570. 6. Aloka PJ. Effect of Gender on Stressful Experiences of First Year Students. An inside from a public university in Kenya. Academicus International Scientific Journal. 2023;14(28):75-88. 7. Amoako M, Amoah-Agyei F, Du C, Fenton JI, Tucker RM. Emotional eating among Ghanaian university students: associations with physical and mental health measures. Nutrients. 2023 Mar 22;15(6):1526. 8. Anyolitho MK, Kabunga A, Anyayo LG, Nakaziba R. Mental health during COVID-19 pandemic: Does social support count? A cross-sectional survey among Lira University students, Northern Uganda. 9. Ayinde, O.O., Akinnuoye, E.R., Molodynski, A., Battrick, O. and Gureje, O., 2022. A descriptive study of mental health and burnout among Nigerian medical students. International Journal of Social Psychiatry, 68(6), pp.1223-1231. 10. Bantjes J, Kazdin AE, Cuijpers P, Breet E, Dunn-Coetzee M, Davids C, Stein DJ, Kessler RC. A web-based group cognitive behavioral therapy intervention for symptoms of anxiety and depression among university students: open-label, pragmatic trial. JMIR mental health. 2021 May 27;8(5):e27400. 11. Bantjes J, Kessler M, Lochner C, Breet E, Bawa A, Roos J, Davids C, Muturiki M, Kessler RC, Stein DJ. The mental health of university students in South Africa: Results of the national student survey. Journal of Affective Disorders. 2023 Jan 15;321:217-26. 12. Bantjes J, Swanevelder S, Jordaan E, Sampson NA, Petukhova MV, Lochner C, Stein DJ, Kessler RC. COVID-19 and common mental disorders among university students in South Africa. South African Journal of Science. 2023 Feb;119(1-2):1-0. 13. Bhagwan R. Insights into the effects of the Covid-19 pandemic on postgraduate students at a university of technology. Perspectives in Education. 2023 Dec 1;41(4):312-28. 14. Chinene B, Mpezeni L, Mudadi L. Mental health literacy of undergraduate radiography students in Zimbabwe. Journal of Medical Imaging and Radiation Sciences. 2023 Dec 1;54(4):662-9. 15. Daudi E, Lyakurwa SE, Amani J. Predictors of psychological help seeking intentions among the university students in Tanzania. International Journal for the Advancement of Counselling. 2023 Mar;45(1):18-37. 16. Davis EJ, Amorim G, Dahn B, Moon TD. Perceived ability to comply with national COVID-19 mitigation strategies and their impact on household finances, food security, and mental well-being of medical and pharmacy students in Liberia. PLoS One. 2021 Jul 9;16(7):e0254446. 17. De Jager S. Connection, desperation and disillusionment: Exploring student wellbeing at a university in South Africa during the COVID-19 pandemic. Perspectives in Education. 2023 Mar 1;41(1):38-55. 18. Dey NE, Oti-Boadi M, Malm E, Selormey RK, Ansah KO. Fear of COVID-19, perceived academic stress, future anxiety, and psychological distress of Ghanaian university students: a serial mediation examination. Journal of Psychology in Africa. 2022 Sep 3;32(5):423-30. 19. Eseadi C. An Online Counseling Intervention for Nigerian Undergraduates with Academic Burnout. Online Submission. 2022;6(1):35-44. 20. Gericke F, Ebert DD, Breet E, Auerbach RP, Bantjes J. A qualitative study of university students' experience of internet‐based CBT for depression. Counselling and Psychotherapy Research. 2021 Dec;21(4):792-804. 21. Gbollie EF, Bantjes J, Jarvis L, Swandevelder S, Du Plessis J, Shadwell R, Davids C, Gerber R, Holland N, Hunt X. Intention to use digital mental health solutions: A cross-sectional survey of university students attitudes and perceptions toward online therapy, mental health apps, and chatbots. Digital Health. 2023 Dec;9:20552076231216559. 22. Hagan Jr JE, Quansah F, Ankomah F, Agormedah EK, Srem-Sai M, Schack T. Evaluating the moderating role of information seeking platforms on university students' risk perception and anxiety during the COVID-19 pandemic in Ghana. Frontiers in Communication. 2023 Mar 22;8:1035593. 23. Idowu OM, Adaramola OG, Aderounmu BS, Olugbamigbe ID, Dada OE, Osifeso AC, Ogunnubi OP, Odukoya OO. A gender comparison of psychological distress among medical students in Nigeria during the Coronavirus pandemic: A cross-sectional survey. African Health Sciences. 2022 Apr 29;22(1):541-0. 24. Johnson FA, Ogunsanmi L, Ayokanmi I (2021) Risk factors for suicidal ideation and self-harm among undergraduate students in a private university in Ogun State, Nigeria. Afr J Nurs Midwifery 4(6):29–42. https:// doi. org/ 10.52589/ AJHNM- 02IMT TGX 25. Kaggwa, M.M., Arinaitwe, I., Muwanguzi, M., Nduhuura, E., Kajjimu, J., Kule, M., Najjuka, S.M., Nkola, R., Ajuna, N., Wamala, N.K. and Machacha, I., 2022. Suicidal behaviours among Ugandan university students: a cross-sectional study. BMC psychiatry, 22(1), p.234. 26. Kaggwa, M.M., Muwanguzi, M., Nduhuura, E., Kajjimu, J., Arinaitwe, I., Kule, M., Najjuka, S.M. and Rukundo, G.Z., 2021. Suicide among Ugandan university students: evidence from media reports for 2010–2020. BJPsych International, 18(3), pp.63-67. 27. Kaggwa MM, Arinaitwe I, Nduhuura E, Muwanguzi M, Kajjimu J, Kule M, Ajuna N, Machacha I, Nkola R, Najjuka SM, Wamala NK. Prevalence and factors associated with depression and suicidal ideation during the COVID-19 pandemic among university students in Uganda: A cross-sectional study. Frontiers in Psychiatry. 2022 Apr 14;13:842466. 28. Khan F. Thinking with Nancy Fraser in Understanding Students’ Experiences of Accessing Psycho-social and Academic Support during the COVID-19 Pandemic. Journal of Student Affairs in Africa. 2022;10(2):1-6. 29. Kihumuro RB, Kaggwa MM, Kintu TM, Nakandi RM, Muwanga DR, Muganzi DJ, Atwau P, Ayesiga I, Najjuma JN, Ashaba S. Knowledge, attitude and perceptions of medical students towards mental health in a university in Uganda. BMC Medical Education. 2022 Oct 20;22(1):730. 30. Kihumuro RB, Kaggwa MM, Nakandi RM, Kintu TM, Muwanga DR, Muganzi DJ, Atwau P, Ayesiga I, Acai A, Najjuka SM, Najjuma JN. Perspectives on mental health services for medical students at a Ugandan medical school. BMC Medical Education. 2022 Oct 25;22(1):734. 31. Kukoyi O, Orok E, Oluwafemi F, Oni O, Oluwadare T, Ojo T, Bamitale T, Jaiyesimi B, Iyamu D. Factors influencing suicidal ideation and self-harm among undergraduate students in a Nigerian private university. Middle East current psychiatry. 2023 Jan 5;30(1):1. 32. Ladi-Akinyemi TW, Okpue AP, Onigbinde OA, Okafor IP, Akodu B, Odeyemi K. Depression and suicidal ideation among undergraduates in state tertiary institutions in Lagos Nigeria. PLoS one. 2023 Apr 26;18(4):e0284955. 33. Losioki B, Mdee H. Perceived psychosocial impacts of COVID-19 among students in higher learning institutions in Dar-es-Salaam, Tanzania. 34. Makgahlela, M., Mothiba, T. M., Mokwena, J. P., & Mphekgwana, P. (2021). Measures to enhance student learning and well-being during the COVID-19 pandemic: Perspectives of students from a historically disadvantaged university. Education Sciences, 11(5), 212. http://dx.doi.org/10.3390/educsci11050212. 35. Makhubela, M., 2021. Comorbid anxiety and depression psychopathology in university students: a network approach. South African Journal of Psychology, 51(1), pp.35-53. 36. Masuku AS, Sibiya MN, Hlengwa RT, Haniff N. The psychological effects of the Coronavirus Disease 2019 (COVID-19) pandemic on students at a University of Technology in KwaZulu-Natal. Journal of Student Affairs in Africa. 2023 Aug 14;11(1). 37. Mekonnen GT, Beyera GK, Tulu A, Roba TT. Perceived influence of COVID-19 pandemic on university students' learning and mental health in Ethiopia. Quality & Quantity. 2023 Jun;57(3):2545-62. 38. Mhata NT, Ntlantsana V, Tomita AM, Mwambene K, Saloojee S. Prevalence of depression, anxiety and burnout in medical students at the University of Namibia. South African Journal of Psychiatry. 2023;29(1). 39. Mohangi K, Olivier H. Counselling Support for Postgraduate Open and Distance e-Learning Students in South Africa: A Case Study. Transformation in Higher Education. 2023;8:265. 40. Mudau T. An exploratory study to understand the mental health care needs of university students (Doctoral dissertation, University of the Witwatersrand, Johannesburg). 41. Mutinta G. Mental distress among university students in the Eastern Cape Province, South Africa. BMC psychology. 2022 Aug 18;10(1):204. 42. Mutiso VN, Ndetei DM, N. Muia E, Musyimi C, Osborn TL, Kasike R, Onsinyo L, Mbijjiwe J, Karambu P, Sounders A, Weisz JR. Prevalence and perception of substance abuse and associated economic indicators and mental health disorders in a large cohort of Kenyan students: towards integrated public health approach and clinical management. BMC psychiatry. 2022 Mar 17;22(1):191. 43. Najjuka SM, Checkwech G, Olum R, Ashaba S, Kaggwa MM. Depression, anxiety, and stress among Ugandan university students during the COVID-19 lockdown: an online survey. African Health Sciences. 2021;21(4):1533-43. 44. Nantaayi B, Ndawula RK, Musoke P, Ssewante N, Nakyagaba L, Wamala JN, Makai EA, Wannyana B, Wamala NK, Kanyike AM, Akech GM. Psychological distress and access to mental health services among undergraduate students during the COVID-19 lockdown in Uganda. Frontiers in Psychiatry. 2022 Jun 2;13:792217. 45. Ndaba N. Exploring first year male students lived experiences of depressive symptoms at the North-West University (Masters dissertation, North-West University (South-Africa)). 46. Ndaba N, Naidoo D, Govender P, van Heerden N, Heaver J, Rambhuron S, Shandu B, Khan F, Ndlovu I. Experiences of online occupational therapy education during the COVID-19 pandemic at a South African university. South African Journal of Occupational Therapy. 2023 Aug;53(2):55-63. 47. Ngcobo ZP. Mental Health Problems and Perceived Barriers in Seeking Psychological Help: A Survey of Students at the University of the Witwatersrand. 48. Niba Rawlings N, Akwah EA, Musisi J, Awanchiri K, Babirye R, Emalieu D, Nduhukyire L, Kakeeto R, Ngongalah L. Perceived risks of COVID-19, attitudes towards preventive guidelines and impact of the lockdown on students in Uganda: A cross-sectional study. PLoS One. 2022 Apr 4;17(4):e0266249. 49. Odiase E. IMPACT OF PSYCHOLOGICAL DISTRESS ON ACADEMIC PERFORMANCE OF FEMALE STUDENTS IN TERTIARY INSTITUTIONS IN LAGOS STATE. 50. Ojewale LY. Psychological state, family functioning and coping strategies among undergraduate students in a Nigerian University during the COVID-19 lockdown. Journal of preventive medicine and hygiene. 2021 Jun;62(2):E285. 51. Onwuegbuzie AJ, Ojo EO, Burger A, Crowley T, Adams SP, Bergsteedt BJ. Challenges Experienced by Students at Stellenbosch University that Hinder their ability Successfully to learn Online during the COVID-19 era: A Demographic and Spatial Analysis. International Journal of Multiple Research Approaches. 2020 Sep 1;12(3). 52. Ossai EN, Eze II, Onyenakazi RC, Ugebe E, Eze B, Obasi O. How large is the burden of depression in a medical school? A cross-sectional study among medical students in Nigeria. Pan African Medical Journal. 2021 Oct 1;40(1). 53. Otanga H, Tanhan A, Musılı PM, Arslan G, Buluş M. Exploring college students’ biopsychosocial spiritual wellbeing and problems during COVID-19 through a contextual and comprehensive framework. International journal of mental health and addiction. 2022 Feb;20(1):619-38. 54. Padmanabhanunni A, Pretorius TB, Isaacs SA. We are not islands: The role of social support in the relationship between perceived stress during the COVID-19 pandemic and psychological distress. International Journal of Environmental Research and Public Health. 2023 Feb 11;20(4):3179. 55. Reta Y, Ayalew M, Yeneabat T, Bedaso A. Social anxiety disorder among undergraduate students of Hawassa university, college of medicine and health sciences, Ethiopian. Neuropsychiatric Disease and Treatment. 2020;16:571-577. Available:http://doi.org/102147/NDT/S235416 56. Rex DL. Perceived social support and sense of coherence as Predictors of psychological well-being amongst university Students during a pandemic. 57. Ross AJ. Impact of COVID-19–Experiences of 5th year medical students at the University of KwaZulu-Natal. South African family practice. 2022 Jun 14;64(3). 58. Seboka, B. T., Hailegebreal, S., Negash, M., Mamo, T. T., Ewune, H. A., Gilano, G., Yehualashet, D. E., Gizachew, G; Demeke, A. D., Worku, A., Endashaw, H., Kassawe,C., Amede, E. S., Kassa, R., & Tesfa, G. A. (2022). Predictors of mental health literacy and information seeking behavior toward mental health among university students in resource-limited settings. International Journal of General Medicine, 15, 8159-8172. 59. Shah SS, Laving A, Okech-Helu VC, Kumar M. Depression and its associated factors: perceived stress, social support, substance use and related sociodemographic risk factors in medical school residents in Nairobi, Kenya. BMC psychiatry. 2021 Dec;21:1-5. 60. Shitandi OB, Efe JO, Sunday IP. Impact of COVID-19 on the mental health of Delta State University students, Nigeria. Acta Bio Medica: Atenei Parmensis. 2021;92(4). 61. Simegn W, Dagnew B, Yeshaw Y, Yitayih S, Woldegerima B, Dagne H. Depression, anxiety, stress and their associated factors among Ethiopian University students during an early stage of COVID-19 pandemic: An online-based cross-sectional survey. PloS one. 2021 May 28;16(5):e0251670. 62. Sserunkuuma J, Kaggwa MM, Muwanguzi M, Najjuka SM, Murungi N, Kajjimu J, Mulungi J, Kihumuro RB, Mamun MA, Griffiths MD, Ashaba S. Problematic use of the internet, smartphones, and social media among medical students and relationship with depression: An exploratory study. Plos one. 2023 May 26;18(5):e0286424. 63. Sumbane GO, Makua NM. Exploring the mental health challenges and coping behaviour of lesbian, gay, and bisexual students at an institution of higher learning. International journal of environmental research and public health. 2023 Mar 1;20(5):4420. 64. Tadesse AW, Mihret ST, Biset G, Kassa AM. Psychological problems and the associated factors related to the COVID-19 pandemic lockdown among college students in Amhara Region, Ethiopia: a cross-sectional study. BMJ open. 2021 Sep 1;11(9):e045623. 65. Van de Velde S, Buffel V, Van Der Heijde C, Çoksan S, Bracke P, Abel T, Busse H, Zeeb H, Rabiee-Khan F, Stathopoulou T, Van Hal G. Depressive symptoms in higher education students during the first wave of the COVID-19 pandemic. An examination of the association with various social risk factors across multiple high-and middle-income countries. SSM-population health. 2021 Dec 1;16:100936. 66. Van Wyk TN. Relationship between social network usage and mental health of first-year students in a South African university (Doctoral dissertation, North-West University (South-Africa)). 67. Visser M, Law-van Wyk E. University students’ mental health and emotional wellbeing during the COVID-19 pandemic and ensuing lockdown. South African Journal of Psychology. 2021 Jun;51(2):229-43. |
| --- |
